# Cytoreductive Surgery Plus Hyperthermic Intraperitoneal Chemotherapy for Peritoneal Metastases From a Small Bowel Adenocarcinoma: Multi-Institutional Experience

**DOI:** 10.1245/s10434-018-6369-x

**Published:** 2018-02-26

**Authors:** Yang Liu, Yutaka Yonemura, Edward A. Levine, Olivier Glehen, Diane Goere, Dominique Elias, David L. Morris, Paul H. Sugarbaker, Jean J. Tuech, Peter Cashin, John D. Spiliotis, Ignace de Hingh, Wim Ceelen, Joel M. Baumgartner, Pompiliu Piso, Kanji Katayama, Marcello Deraco, Shigeki Kusamura, Marc Pocard, François Quenet, Sachio Fushita, The BIG-RENAPE Group, Frédéric Marchal, Pablo Ortega-Deballon

**Affiliations:** 1 NPO to Support Peritoneal Surface Malignancy Treatment, Kyoto, Japan; 2 Peritoneal Dissemination Center, Kishiwada Tokushukai Hospital, Osaka, Japan; 3 Peritoneal Dissemination Center, Kusatsu General Hospital, Shiga, Japan; 4 Wake Forest University Baptist Medical Center, Winston-Salem, NC USA; 5 Centre Hospitalo-Universitaire Lyon Sud, Hospices Civils de Lyon, Pierre, France; 6 Institut Gustave Roussy Cancer Center, Villejuif, France; 7 University of New South Wales, St George Hospital, Sydney, Australia; 8 Washington Cancer Institute, Washington Hospital Center, Washington DC, USA; 9 Centre Hospitalier de Rouen, Rouen, France; 10 Department of Surgical Sciences, Colorectal Surgery, Uppsala University, Uppsala, Sweden; 11 Department of Surgical Oncology, Metaxa Cancer Memorial Hospital, Pireus, Greece; 12 Catharina Hospital, Eindhoven, The Netherlands; 13 Department of Gastrointestinal Surgery, Ghent University Hospital, Ghent, Belgium; 14 Division of Surgical Oncology of Moores Cancer Center, University of California San Diego, San Diego, USA; 15 Krankenhaus Barmherzige Brueder Regensburg, Regensburg, Germany; 16 Cancer Care Promotion Center, Medical School Hospital, University of Fukui, Fukui, Japan; 17 Department of Surgery, National Cancer Institute, Milan, Italy; 18 Hopital Lariboisière, Assistance Publique Hôpitaux de Paris, Paris, France; 19 Centre Val D’Aurelle, Montpellier, France; 20 Department of Surgery, Kanazawa University Hospital, Kanazawa University, Kanazawa, Japan

## Abstract

**Background:**

The multi-institutional registry in this study evaluated the outcome after cytoreductive surgery (CRS) plus hyperthermic intraperitoneal chemotherapy (HIPEC) for patients with peritoneal metastases (PM) from small bowel adenocarcinoma (SBA).

**Methods:**

A multi-institutional data registry including 152 patients with PM from SBA was established. The primary end point was overall survival (OS) after CRS plus HIPEC.

**Results:**

Between 1989 and 2016, 152 patients from 21 institutions received a treatment of CRS plus HIPEC. The median follow-up period was 20 months (range 1–100 months). Of the 152 patients, 70 (46.1%) were women with a median age of 54 years. The median peritoneal cancer index (PCI) was 10 (mean 12; range 1–33). Completeness of cytoreduction (CCR) 0 or 1 was achieved for 134 patients (88.2%). After CRS and HIPEC, the median OS was 32 months (range 1–100 months), with survival rates of 83.2% at 1 year, 46.4% at 3 years, and 30.8% at 5 years. The median disease-free survival after CCR 0/1 was 14 months (range 1–100 months). The treatment-related mortality rate was 2%, and 29 patients (19.1%) experienced grades 3 or 4 operative complications. The period between detection of PM and CRS plus HIPEC was 6 months or less (*P* = 0.008), and multivariate analysis identified absence of lymph node metastasis (*P* = 0.037), well-differentiated tumor (*P* = 0.028), and PCI of 15 or lower (*P* = 0.003) as independently associated with improved OS.

**Conclusion:**

The combined treatment strategy of CRS plus HIPEC achieved prolonged survival for selected patients who had PM from SBA with acceptable morbidity and mortality.

Small bowel cancer is a rare malignancy comprising less than 5% of all gastrointestinal cancers.^1^ In the United States, about 9410 patients received a new diagnosis of small bowel cancer in 2015.^2^ Adenocarcinoma is a frequent subtype, accounting for 37% of all small bowel cancers.^1^ Clinicians find it challenging to detect small bowel adenocarcinoma (SBA) in early stages of cancer due to vague or even absent symptoms and lack of a screening examination. Therefore, SBA typically presents as advanced disease.^3^

Surgical resection remains the mainstay of treatment strategy for patients with SBA. However, the prognosis of patients with SBA is poor, with a 5-year survival rate of 15–33% and a median overall survival (OS) ranging from 12 to 20 months.^1^,^3^–^5^

Peritoneal metastases (PM) and hepatic metastases are the most common failure patterns for SBA.^4^ The current standard treatment for patients with advanced SBA is systemic chemotherapy, with regimens typically extrapolated from those for colorectal cancer.^5^ In a prospective phase 2 study, advanced SBA patients who received chemotherapy including capecitabine and oxaliplatin had a median OS of 20.4 months.^6^ In two multicenter retrospective studies reported by Zaanan et al.^7^ and Tsushima et al.^8^ advanced SBA patients who received chemotherapy using FOLFOX and fluoropyrimidine-oxaliplatin had OS periods of 17.8 months and 22.2 months, respectively, which were significantly better than the OS for patients who received other chemotherapy regimens. As a result, fluoropyrimidine-oxaliplatin is now considered as a first-line chemotherapy regimen for advanced SBA. However, a consensus on the treatment for SBA patients with PM has not been reached.

Cytoreductive surgery (CRS) plus hyperthermic intraperitoneal chemotherapy (HIPEC) has been widely applied in the treatment of PM from various origins such as colorectal cancer, malignant peritoneal mesothelioma, and pseudomyxoma peritonei.^9^–^11^ Moreover, the survival benefit for selected patients has been proved. Several retrospective single-institution studies evaluating CRS plus HIPEC in the treatment of PM from SBA have been reported.^12^–^18^ In general, the patient numbers in these studies are very low, prohibiting adequate analysis of efficacy and safety.

Therefore, in an effort to collect sufficient data to evaluate CRS plus HIPEC for patients with PM from SBA, a multi-institutional study was performed including all consecutive cases in participating centers.

## Methods

A multi-institutional data registry on PM from SBA treated by CRS plus HIPEC was established during the 9th International Congress on Peritoneal Surface Malignancy at Amsterdam, the Netherlands, in October 2014. Ethics approval was obtained from the participating institutions through their institutional review boards or through the chairpersons of their ethics committees.

The inclusion criteria specified histologic confirmation of PM from SBA and reception of treatment involving CRS plus HIPEC. The exclusion criteria ruled out patients with PM from small bowel cancer with a histology of carcinoid, lymphoma, gastrointestinal stromal tumors, and sarcoma as well as patients who did not receive treatment of CRS plus HIPEC.

The patients were treated with CRS including the peritonectomy procedures as indicated by Sugarbaker.^19^ During the surgery, the extent of PM via the peritoneal cancer index (PCI) was evaluated detail.^20^

After CRS, HIPEC was administered using an open coliseum procedure or closed technique, depending on the individual unit’s preference, with chemotherapy agents in heated solution. The extent of CRS was determined by completeness of cytoreduction (CCR) according to the criteria described by Surgarbaker.^20^ Adverse events occurring during the 3 months after surgery were graded according to the National Cancer Institute Common Terminology Criteria for Adverse Events, version 3.0.^21^

A standard data form was created to retrieve relevant information on the course of patients with PM from SBA treated by CRS plus HIPEC. Clinicopathologic and treatment-related variables were included in the subsequent data analysis to identify prognostic factors because they possibly held potential clinical implications for future patient management.

### Statistical Analysis

Overall survival after CRS plus HIPEC was calculated from the date of CRS plus HIPEC to the patient’s death or the latest follow-up visit. The primary end point of this study was the OS after CRS plus HIPEC. The secondary end points were identification of the clinicopathologic and treatment-related prognostic factors for OS and evaluation of the safety of CRS plus HIPEC.

Disease-free survival (DFS) was calculated from the date of CRS plus HIPEC to the date of recurrence detected in patients who received complete cytoreduction of CCR 0/1. Survival analysis was performed using the Kaplan–Meier method and compared using the log-rank test and a Cox proportional hazards regression model using variables with significant *P* values from the univariate analysis for the multivariate analysis. All statistical analyses were performed with the Statistical Package for Social Sciences, version 17.0 (IBM, Armonk, NY, USA), and *P* values lower than 0.05 were considered to be statistically significant.

## Results

### Clinicopathologic Data

Between 1989 and 2016, 152 patients from 21 institutions (17 from Western countries, 4 from Asia) with PM from SBA received a treatment of CRS plus HIPEC. The clinicopathologic characteristics of the patients are listed in Table 1. Of the 152 patients, 70 (46.1%) were women and 82 (53.9%) were men with a median age of 54 years (mean 52.5 ± 11.0 years; range 30–77 years).

**Table 1:** Characteristics of 152 patients with peritoneal metastases (PM) from small bowel adenocarcinoma treated with cytoreductive surgery (CRS) plus hyperthermic intraperitoneal chemotherapy (HIPEC)

Characteristic	Patients (*n*)	%
Age (years)		
≤ 60	113	74.3
> 60	38	25.0
Unknown	1	0.7
Sex		
Male	82	53.9
Female	70	46.1
Area		
Western country	115	75.7
Asia	37	24.3
Time period of CRS+HIPEC		
1989–2001	12	7.9
2001–2010	59	38.8
2011–2016	77	50.7
Unknown	4	2.6
Surgical resection of primary tumor before CRS+HIPEC		
Yes	123	81
No	27	17.7
Unknown	2	1.3
Primary tumor site		
Duodenum	10	6.6
Jejunum	86	56.6
Ileum	44	29.0
Unknown	12	7.8
Tumor differentiation		
Well-differentiated	29	19
Moderately differentiated	72	47.4
Poorly differentiated	38	25
Unknown	13	8.6
Synchronous PC		
Yes	96	63.2
No	51	33.5
Unknown	5	3.3
Lymph node metastasis		
Yes	45	29.6
No	88	57.9
Unknown	19	12.5
Extraperitoneal metastasis		
Yes	13	8.6
No	138	90.7
Unknown	1	0.7
Neoadjuvant chemotherapy before CRS+HIPEC		
Yes	82	54.0
No	57	37.5
Unknown	13	8.5
Presence of ascites		
Yes	23	15.1
No	110	72.4
Unknown	19	12.5
Peritoneal cancer index		
≤ 15	96	63.2
> 15	40	26.3
Unknown	16	10.5
Completeness of cytoreduction		
0	114	75
1	20	13.1
2 or 3	15	9.9
Unknown	3	2.0
Postoperative complication		
No	85	55.9
Yes	60	39.5
Unknown	7	4.6
Adjuvant chemotherapy after CRS+HIPEC		
Yes	81	53.3
No	46	30.2
Unknown	25	16.5

For 123 (81%) of the patients, primary tumor resection was performed before CRS and HIPEC, and for 82 of the patients, systemic chemotherapy was administered between detection of PM and CRS plus HIPEC. The main regimens of preoperative systemic chemotherapy were FOLFOX, FOLFIRI, XELOX, and TS-1. Adjuvant chemotherapy was administered to 81 patients after CRS plus HIPEC. Similarly, FOLFOX, FOLFIRI, and TS-1 were the main adjuvant chemotherapy regimens in this study.

For 51 patients with metachronous PM (33.6%), the median interval between primary surgery and detection of PM was 13 months (mean 17 ± 16.8 months; range 1–70 months). The histology of 10 patients showed a component of mucinous adenocarcinoma. The median interval between detection of PM and CRS plus HIPEC was 5 months (mean 7.4 ± 9.5 months; range 0–60 months). Of 13 patients (8.6%) with extraperitoneal metastasis besides PM, 12 had liver metastasis. The remaining patient had lung metastasis. The median PCI found at CRS and HIPEC was 10 (mean 12; range 1–33).

### Treatment-Related Data

In this study, CCR 0 and 1 were achieved respectively for 114 (75%) and 20 (13.2%) patients. Total parietal peritonectomy, defined as peritonectomy performed in areas including both sides of the anterior abdominal wall as well as the subphrenic area, paracolic gutter, Morison’s pouch, and pelvis, was performed for 46 patients (30.3%), and partial peritonectomy was performed for 78 patients (51.3%). The surgical resections included omentectomy (*n* = 117), small bowel (*n* = 132), colon and/or rectum (*n* = 101), cholecystectomy (*n* = 65), splenectomy (*n* = 62), appendectomy (*n* = 71), hysterectomy (*n* = 35), oophorectomy (*n* = 36), partial hepatectomy (*n* = 15), gastrectomy (*n* = 6), partial pancreatectomy (*n* = 7), and partial cystectomy (*n* = 4).

All 152 patients (100%) underwent HIPEC. The chemotherapy regimens used for HIPEC are summarized in Table 2, with 13 institutions using a coliseum (open) procedure, and 8 units using a closed technique. The duration of HIPEC was 30 to 120 min (median, 60 min), and the intraperitoneal temperature was 41–43 °C (median 42 °C). For 12 patients (7.9%), early postoperative chemotherapy (EPIC) was performed after surgery. The mean duration of CRS plus HIPEC was 380 min (median 360 min; range 60–805 min). The mean volume of blood loss was 0.750 L (median 0.500 L; range 0.020–5.850 L). The mean volume of red blood cell (RBC) transfusion was 2 units (range 0–16 units), with 92 patients receiving no transfusion of RBC. The mean transfusion of fresh frozen plasma (FFP) was 2.5 units (range 0–18 units), with 90 patients receiving no transfusion of FFP. The mean hospital stay was 19 days (median 16 days; range 5–84 days). A repeat CRS plus HIPEC was performed for 18 patients (11.8%) after tumor recurrence was detected.

**Table 2:** Chemotherapy agents used in hyperthermic intraperitoneal chemotherapy (HIPEC) after cytoreductive surgery (CRS) for peritoneal metastases (PM) from small bowel adenocarcinoma (SBA)

Chemotherapy	*n*
MMC regimens	73
MMC	58
MMC+cisplatin	7
MMC+doxorubicin	5
MMC+irinotecan	3
Oxaliplatin regimens	72
Oxaliplatin (± 5-FU/LV)	48
Oxaliplatin+irinotecan	24
Other regimens	7
Doxorubicin	1
Docetaxel+cisplatin	1
Doxorubicin+cisplatin	2
Docetaxel	3

*MMC* mitomycin C, *5-FU* 5- fluorouracil

The mortality rate was 2%, with one patient dying due to multiple organ failure 35 days after surgery, one patient dying due to disseminated intravascular coagulation 49 days after surgery, and one patient dying due to pulmonary failure 84 days after surgery.

The overall morbidity rate was 39.5% (7 unknown cases), with 29 patients (19.1%) experiencing major complications of grade 3 or 4. For 10 patients (6.6%), a reoperation was needed after CRS plus HIPEC. The major complications were intraperitoneal abscess (*n* = 8), pleural effusion (*n* = 5), septicemia (*n* = 7), intestinal fistula (*n* = 7), hemorrhage (*n* = 6), neutropenia (*n* = 4), ileus (*n* = 4), gastrointestinal bleeding (*n* = 2), wound dehiscence (*n* = 3), and urinary bladder fistula (*n* = 2).

### Survival Outcome

**Fig. 1:**
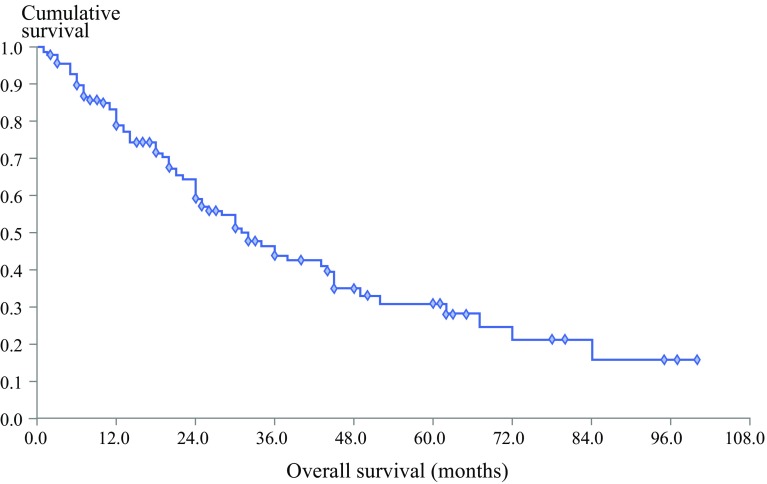
Overall survival after cytoreductive surgery plus hyperthermic intraperitoneal chemotherapy for patients with peritoneal metastases from small bowel adenocarcinoma (*n* = 152)

**Fig. 2:**
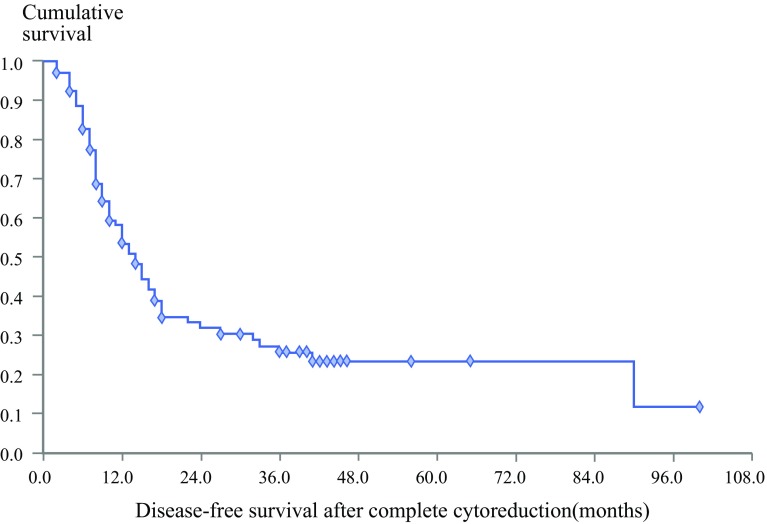
Disease-free survival of patients who received complete cytoreduction (CC0/1) plus hyperthermic intraperitoneal chemotherapy (*n* = 134)

For all 152 patients who received CRS plus HIPEC, the median follow-up period was 20 months (range 1–100 months). The median OS after CRS plus HIPEC was 32 months (range 1–100 months). After CRS plus HIPEC, the survival rate was 83.2% at 1 year, 46.4% at 3 years, and 30.8% at 5 years (Fig. 1). The median DFS after CRS plus HIPEC for patients who received CCR 0 or 1 was 14 months (range 1–100 months) (Fig. 2). Until the last follow-up visit, 47 patients were alive without evidence of disease, and 26 patients were alive with disease.

Univariate analysis identified the following 13 significant prognostic variables associated with improved survival after CRS plus HIPEC: resection of primary tumor before CRS plus HIPEC (*P* = 0.045), interval of 6 months or less between detection of PM and CRS plus HIPEC (*P* = 0.008), well-differentiated tumor (*P* = 0.037), absence of lymph node metastasis during CRS plus HIPEC (*P* < 0.0001), absence of extraperitoneal metastasis (*P* = 0.030), normal value of CA 125 (*P* = 0.028), normal value of CA 19-9 (*P* = 0.008), absence of ascites (*P* = 0.022), PCI of 15 or lower (*P* < 0.0001), CCR of 0 (*P* < 0.0001), oxaliplatin-based regimen of HIPEC (*P* = 0.038), absence of postoperative complications (*P* = 0.022), and performance of a repeat CRS plus HIPEC after detection of recurrence (*P* = 0.03) (Table 3).

**Table 3:** Univariate analysis of overall survival after cytoreductive surgery (CRS) plus hyperthermic intraperitoneal chemotherapy (HIPEC) for patients with peritoneal metastases (PM) from small bowel adenocarcinoma (SBA)

Variable	Median survival (months)	95% CI	Log-rank *P* value
Age (years)			
≤ 60	32	22.1–41.9	0.928
> 60	30	22.0–38.0	
Sex			
Male	34	26.3–35.7	0.528
Female	30	18.0–50.0	
Area			
Western countries	30	21.6–38.4	0.404
Asia	36	19.3–52.7	
Time period of CRS and HIPEC			
1989–2000	42	2.93–87.1	
2001–2010	25	19.2–28.8	0.066
2011–2016	–	–	
Resection of primary tumor			
Yes	34	21.3–46.7	0.045
No	24	21.1–26.9	
Primary tumor site			
Duodenum	30	41.7–55.8	0.402
Jejunum	38	21.8–54.2	
Ileum	28	15.7–40.3	
Tumor differentiation			
Well-differentiated tumor	54	1.6–106.4	0.037
Moderately differentiated tumor	32	19.5–44.5	
Poorly differentiated tumor	24	17.3–30.7	
Synchronous PC			
Yes	30	21.2–38.8	0.333
No	36	20.9–51.1	
Interval between detection of PM and CRS+HIPEC (months)			
≤ 6	36	21.1–50.9	0.008
> 6	14	5.8–22.2	
Lymph node metastasis			
Yes	18	10.6–25.4	< 0.001
No	36	24.1–47.9	
Extraperitoneal metastasis			
Yes	20	10.3–29.7	0.030
No	32	18.8–45.2	
Chemotherapy before CRS			
Yes	30	20.1–39.9	0.742
No	30	18.3–41.7	
Abnormal CA125			
Yes	25	0–53.2	0.028
No	43	23.7–62.3	
Abnormal CA 19-9			
Yes	21	11.8–30.2	0.008
No	36	20.6–51.4	
Presence of ascites			
Yes	24	0–48.1	0.022
No	34	20.7–47.3	
Peritoneal cancer index			
≤ 15	67	26.1–107.9	< 0.001
> 15	18	10.9–25.1	
Completeness of cytoreduction			
0	43	34.2–51.8	< 0.001 (CCR 0 vs 1–3)
1	24	18.8–29.2	
2 or 3	6	4.4–7.6	
Chemotherapy regimen of HIPEC			
MMC-based regimen	25	19.0–31.0	0.038
Oxaliplatin-based regimen	49	15.6–82.4	
Postoperative complications			
No	44	33.7–54.3	0.022
Yes	24	19.8–28.2	
Adjuvant chemotherapy after CRS			
Yes	34	26.2–41.8	0.167
No	20	5.6–34.4	
Performance of a repeat CRS and HIPEC			
Yes	44	28.6–58.4	0.030
No	26	20.5–31.5	

*CI* confidence interval, *MMC* mitomycin C

Other variables such as age, sex, area, time period of CRS plus HIPEC, primary tumor site, synchronous PM, neoadjuvant chemotherapy, adjuvant chemotherapy, and delivery details of HIPEC were not found significantly associated with OS after CRS plus HIPEC. Moreover, the univariate analysis found absence of lymph node metastasis (*P* = 0.029), normal value of CA19-9 before CRS plus HIPEC (*P* = 0.001), absence of acites (*P* = 0.021), PCI of 15 or lower (*P* = 0.009), and absence of postoperative complications (*P* = 0.001) to be associated significantly with improved DFS after CCR 0 or 1.

A multivariate analysis with a Cox regression model was performed to determine independent predictors of improved OS after CRS plus HIPEC. An improved OS after CRS plus HIPEC was predicted by an interval of 6 months or less between detection of PM and date of CRS plus HIPEC [hazard ratio(HR) 0.180; 95% confidence interval (CI) 0.089–0.697; *P* = 0.008], no lymph node metastasis during CRS plus HIPEC (HR 0.315; 95% CI 0.138–0.941; *P* = 0.037), well-differentiated tumor (HR 0.052; 95% CI 0.020–0.801; *P* = 0.028), and a PCI of 15 or lower (HR 0.002; 95% CI 0.000–0.104; *P* = 0.003).

## Discussion

The survival of patients with advanced SBA is poor, with a median overall 5-year survival rate of 3–5%.^5^,^22^ In addition, a median survival of approximately 20 months is reported for patients treated with oxaliplatin-based chemotherapy.^6^,^23^

In the current study, SBA patients who had PM treated with CRS plus HIPEC experienced a median OS of 32 months and a 5-year survival rate of 30.8%, reaching the median OS obtained for patients who had colorectal carcinomatosis treated with the same therapeutic strategy.^24^ This promising result suggests that CRS plus HIPEC may confer a promising survival benefit for patients with PM from SBA. Moreover, an interval of 6 months or less between detection of PM and date of CRS plus HIPEC is recommended because it was identified as an independent predictor for better OS in the current study.

Although details of the method for delivering HIPEC varied among the institutions in this study, they were not associated with OS. Oxaliplatin-based chemotherapy was not identified as an independent variable, but showed a significant survival advantage over the mitomycin C (MMC)-based chemotherapy regimen in the univariable analysis in this registry. In addition, considering the survival advantage of oxaliplatin-based chemotherapy over other chemotherapy regimens demonstrated by other studies,^6^–^8^ we suggest recommending an oxaliplatin-based chemotherapy regimen for HIPEC for patients with PM from SBA. However, it is worth noting that the numbers of patients treated with various regimens precludes definitive conclusions on the optimal agent in the perfusate.

In the current study, well-differentiated tumor, absence of lymph node metastasis, and a PCI of 15 or lower were independently associated with improved OS. These factors also were demonstrated to have a favorable influence on the survival of patients with SBA in other retrospective studies.^24^–^28^ Patients with well-differentiated tumor had a median OS of 54 months, which was significantly better than the OS of patients with moderately or poorly differentiated tumor.

Lymph node metastasis was frequent in SBA patients with PM, at an incidence of 33% and even 48.3% during the whole disease course in this study. The median OS after CRS plus HIPEC was significantly better for the patients without lymph node metastasis than for the patients with lymph node metastasis (36 vs 18 months). Although severe tumor burden also is usually demonstrated with strong association to poorer survival for patients with PM,^7^–^9^ it generally is difficult to obtain precise details of intraperitoneal tumor dissemination until CRS.

Recently, laparoscopic HIPEC has been used for precise understanding and reduction of PCI before CRS in gastric cancer.^29^ By performing laparoscopic HIPEC before CRS, tumor dissemination can be directly understood, and PCI can be significantly decreased at the same time. Therefore, laparoscopic HIPEC can be considered with preoperative systemic chemotherapy for PM from SBA.

In the registry of this study, the patients who received CCR of 0 had a median OS of 43 months, which was significantly better than the OS for patients who received CCR 1–3 surgery (*P* < 0.001).

The feasibility of achieving complete cytoreduction depends mainly on tumor burden and technique expertise. For patients with severe tumor burden, achieving a CCR of 0 may increase the risk of postoperative major complications. Postoperative complication was related to postoperative OS and DFS. Although almost all the patients had disease recurrence during the long-term follow-up assessment, those selected to undergo a repeat CRS plus HIPEC had better survival. However, only a minority of the patients received a repeat CRS plus HIPEC. As a result, an attempt should be made to avoid postoperative complication during CRS plus HIPEC, and close follow-up evaluation should be carried out after complete cytoreduction to detect potentially resectable recurrence, thereby maximizing the chance of repeat CRS and HIPEC.

Regarding neoadjuvant and adjuvant chemotherapy, no significant difference in survival was shown in this registry. However, systemic chemotherapy after detection of PM may contribute to a decrease in the values of CA125 and CA 19-9, which were significantly related to postoperative OS. Similarly, the survival benefit of systemic chemotherapy versus best supportive care alone has been shown in several retrospective studies.^22^,^23^ Although the rarity of SBA makes randomized trials impractical for comparing the efficacy of chemotherapy regimens based on the efficacy of the fluoropyrimidine-oxaliplatin-based chemotherapy regimen reported in multicenter retrospective studies, neoadjuvant and adjuvant systemic chemotherapy using the fluoropyrimidine-oxaliplatin-based regimen can be considered as an option for patients with PM from SBA.^7^,^8^ Moreover, the neoadjuvant course of therapy would have to be limited so CRS and HIPEC can be completed within 6 months after a PM diagnosis.

Aparicio et al.^23^ studied the molecular biology of SBA and showed that defective mismatch repair (dMMR) phenotype and mutated KRAS status were significantly associated with improved OS for all patients and for stage 4 patients, respectively. The progress in molecular characterization and pathogenesis of SBA may have potential for prospective development of novel targeted therapies.^20^

In conclusion, the large registry in this study demonstrated that treatment using CRS plus HIPEC achieved prolonged survival for selected patients with PM from SBA and showed acceptable safety. Therefore, CRS plus HIPEC should be considered as a new treatment option for selected patients with PM from SBA. Based on the reported data, a consensus statement by the Peritoneal Surface Oncology Group International (PSOGI) with a clear recommendation for a uniform HIPEC protocol for adenocarcinoma of the small bowel should be published.
